# Optimization of Cost-Effective and Reproducible Flexible Humidity Sensors Based on Metal-Organic Frameworks

**DOI:** 10.3390/s20236981

**Published:** 2020-12-07

**Authors:** Victor Toral, Florin C. Loghin, Antonio Rodríguez-Diéguez, Alejandro Lapresta-Fernández, Diego P. Morales, Almudena Rivadeneyra, Alfonso Salinas-Castillo

**Affiliations:** 1 Department of Electronics and Computer Technology, University of Granada, 18071 Granada, Spain; vtoral@ugr.es (V.T.); diegopm@ugr.es (D.P.M.); arivadeneyra@ugr.es (A.R.); 2 Institute for Nanoelectronics, Technical University of Munich, 80333 Munich, Germany; florin.loghin@tum.de; 3 Department of Inorganic Chemistry, University of Granada, 18071 Granada, Spain; antonio5@ugr.es; 4 Department of Analytic Chemistry, University of Granada, 18071 Granada, Spain; lapresta@ugr.es

**Keywords:** interdigitated electrodes, metal-organic frameworks, flexible electronics, printed electronics

## Abstract

In this letter, we present the extension of a previous work on a cost-effective method for fabricating highly sensitive humidity sensors on flexible substrates with a reversible response, allowing precise monitoring of the humidity threshold. In that work we demonstrated the use of three-dimensional metal-organic framework (MOF) film deposition based on the perylene-3,4,9,10-tetracarboxylate linker, potassium as metallic center and the interspacing of silver interdigitated electrodes (IDEs) as humidity sensors. In this work, we study one of the most important issues in efficient and reproducible mass production, which is to optimize the most important processes’ parameters in their fabrication, such as controlling the thickness of the sensor’s layers. We demonstrate this method not only allows for the creation of humidity sensors, but it also is possible to change the humidity value that changes the actuator state.

## 1. Introduction

Many industrial processes are sensitive to water content in the air. For this reason, humidity sensors are crucial for several industries, such as electronics or food that need to control relative humidity (RH) during manufacturing or transportation [1,2,3]. Moreover, not only does industry need these sensors, domotic systems or intelligent control in household applications, for example, also require this type of sensor to control and accommodate ambient conditions for comfort. For these needs, lowering the cost and size of these sensors can be a great benefit, especially in this era of massive Internet of Things (IoT) connected devices collecting a huge amount of data that requires a massive distribution of sensors [4].

RH sensors are typically based on ceramic materials such as aluminum oxide, semiconducting materials such as SiO_2_, or polymers [5,6]. Moreover, 1D and 2D materials have appeared as solutions to flexible and highly integrable sensors, as for example carbon nanotubes [7] or silicon nanosheets [8]. In the same context of novel materials, carbon-based materials have been also widely used [9]. Furthermore, in novel miniaturized sensors, the use of a printed layer of different materials like organic compounds or nanoparticles over interdigitated electrodes (IDEs) is common [10,11]. Due to their structural features, metal-organic frameworks (MOFs) have properties that have attracted great interest in recent years [12,13,14,15]. These types of materials can also be used as RH sensors because of their luminescence or impedance [16,17].

Typically, RH sensors have been passive, based on the variation in capacitance or resistance of the materials employed. Several works have treated active devices that react to RH; they are based on the variation of threshold voltage in Field Effect Transistors (FETs) or the resistivity of the channel. However, these devices are indirect measures of RH, as the device does not react to the RH values [18,19]. In a previous work, we demonstrated the potential use of the perylene-3,4,9,10-tetracarboxylate linker with potassium (K-Pery) for humidity actuators [20]. Silver interdigitated electrodes (IDEs) were drop-casted with K-Pery water solution, showing a drastic change in impedance response at 40% RH from capacitive to resistive response. However, it did not cover the influence of the deposition of the K-Pery solution, the IDEs’ dimensions, or if these parameters could help to tune the RH level that triggers the device from capacitive to resistive mode. In this paper, we fabricate similar devices but with more controllable processes and analyze their responses.

We studied the influence of two aspects in the fabrication of sensors: the deposition of K-Pery solution and the interspacing of IDEs. For the deposition of K-Pery, in previous work we employed drop-casting, which despite its simplicity did not allow us to control the thickness of the deposited layer with accuracy. To solve this problem, multiple techniques are available [21,22]. For example, roller coating is widely used in industries with high production volumes; however, it is quite expensive for small volumes of production [23]. Another possibility is spin coating, the same technique used for the fabrication of CD-R photo sensible layers [24]. However, in this case the presence of IDEs could affect the roughness and uniformity of the surface of thin layers. In terms of cost, despite being better than roller coating techniques, it is not the least expensive solution. Spray coating was found to be the most adequate for this application; it is a low-cost technique, and the thickness of the layer is more controllable just by varying the mixed portion of air and coating material [25,26].

The other fabrication parameter that can be studied is the IDEs’ interspacing. As the system detects humidity through conductivity, with a higher interspacing, better conductivity of K-Pery will be needed to have the same conductivity between IDE fingers. This could allow us to control the trigger level for the device.

## 2. Materials and Methods

### 2.1. K-Pery Preparation

The compound was obtained by conventional routes by using reagents from commercial sources as received in a similar way as it was done in the previous work [20]. The perylenetetracarboxylic derivative ligand (0.1 mmol) was added to 5 mL of H_2_O. The resulting red solution was sonicated for 30 min, and then 5 mL of an aqueous solution with KOH (0.1 mmol) was incorporated into it. The resulting solution was heated for 24 h under a 150W IR lamp at 25 cm distance. Orange hexagonal crystals were obtained totally pure, whose composition was C_72_H_36_K_8_O_28_ (See Figure A3 in Supporting Information). The yield was normally close to 65%. 

### 2.2. Sensor Fabrication

For the device fabrication, K-Pery powder was brought into the solution by dissolving it in deionized water (Di H_2_O) in a ratio of 1:10 (K-Pery: Di H_2_O), followed by sonication in an ultrasonic bath for 15 min. The K-Pery powder is a 3D metal-organic framework based on the perylene-3,4,9,10-tetracarboxylate linker and potassium as the metallic center. For the deposition of the K-Pery solution, a handheld airbrush was used. The control parameters were K-Pery solution, airbrush sample spacing and airbrush opening, while the hotplate temperature was varied from 70 °C to 110 °C. These temperatures were selected to generate two scenarios for the evaporation of the solvent at the other fixed process parameters. At 70 °C, evaporation was relatively slow with a visible wet film forming allowing for the merging of droplets, which can be referred to as a wet spray, while at 110 °C the film instantaneously dried without droplets merging. This can be referred to as dry spraying. Table 1 summarizes the combinations of dry scenarios and volume deposited used in this work. The selection of the volume was to cover as much of the available space as possible.

**Figure 1:**
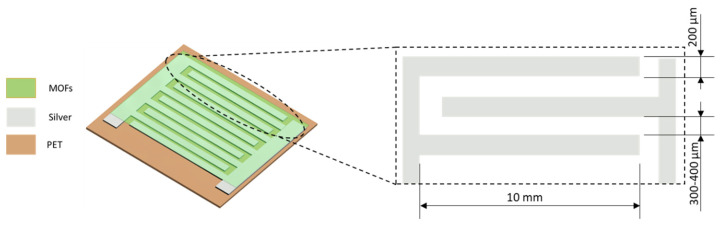
Schematic image of the fabricated sensor and detailed dimensions of the IDE.

After the deposition of the solution onto the substrate, an interdigitated electrode structure was patterned onto the polyethylene terephthalate (PET) substrate. The deposition of the electrodes was done by screen printing a silver conductive paste (Sigma Aldrich, Darmstadt, Germany) with a manual screen printer (Siebdruck Versand). The sensor area was fixed to 25 mm^2^ and a fixed finger width of 200 µm, while for finger spacing, 300 µm and 400 µm were used for comparison. The drying of the Ag layer was performed at 60 °C for 10 min in a UF55 oven from Memmert (Schwabach, Germany). Figure 1 represents schematically the sensor composition and the dimensions of the IDE fabricated. 

### 2.3. Characterization

An optical microscope DM2500 equipped with a DFC295 camera (Leica Microsystems, Germany) was employed for the images.

For the electrical characterization, an impedance analyzer E4990A (KeysightTechnologies, Boeblingen, Germany) in combination with an impedance probe kit (42941A) was controlled with LabView 2016. The interface with the sensor was a SubMiniature version A (SMA) male connector glued with silver paste. The excitation applied was V_DC_ = 0 and V_AC_ = 500 mV in the range of 100 Hz to 10 MHz. Calibration was done to compensate the parasitic elements, as performed previously [27]. To control temperature and humidity, a climatic chamber VCL4006 (Vötsch Industrietechnik GmbH, Balingen, Germany) was used to place the sensor. The monitoring was performed over the climatic sensor system. During experiments, moisture content was ramped up from 20% to 80% in 10% steps with pauses of 1 h to stabilize the value in the whole chamber volume.

## 3. Results

### 3.1. Physical Characterization

**Figure 2:**
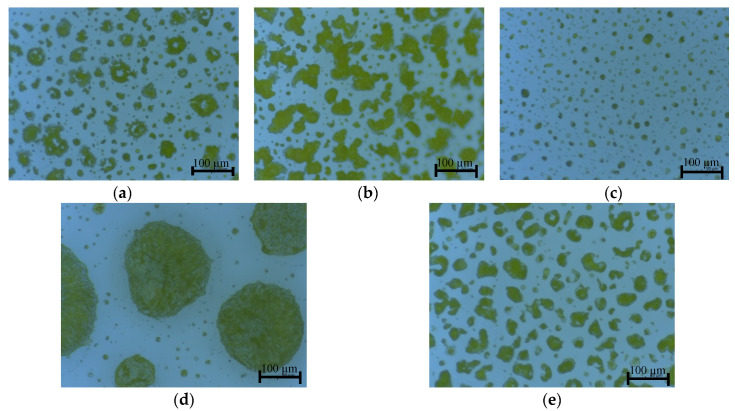
Optical microscope images for the spray coating with the different fabrication parameters: type 1 (**a**); type 2 (**b**); type 3 (**c**); type 4 (**d**); type 5 (**e**).

Optical microscope images of the different types of sprayed K-Pery layers are shown in Figure 2. They show the differences in deposition method, especially in the size and morphology of the crystalized MOFs. Figure 2a displays a clear coffee stain effect that can be attributed to a solvent evaporation time frame that was too long, allowing for such effects to happen. Microscopically the material was transported to the edge of the droplets, leaving void areas. For further reference see [28]. Figure 2c displays a time frame that was too short, as these individual droplets did not merge as the solvents evaporated prior to inter-droplet flow.

### 3.2. Response to Moisture Content

The impedance of the fabricated devices was characterized towards RH at different frequencies. After checking the response of type 1, we discarded it because it did not show a consistent response during consecutive tests, which can be attributed to the fact that these fabrication parameters resulted in a too sparsely deposited film. From now on, the manuscript only presents the characterization data of type 2–5 sensors. 

**Figure 3:**
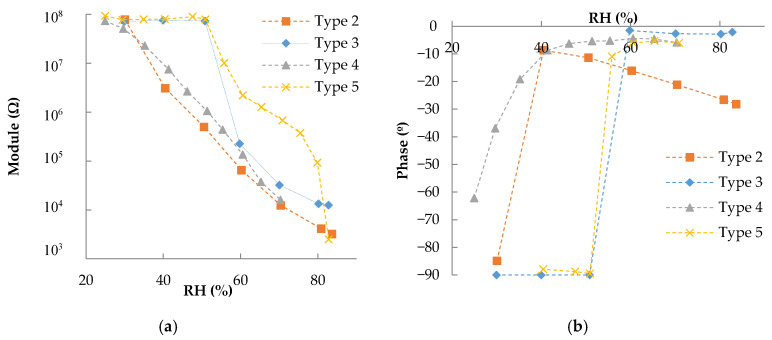
Impedance response towards RH at 100 Hz and 40 °C for the different deposited layers with distance between consecutive fingers of 300 µm: (**a**) module and (**b**) phase.

**Figure A1:**
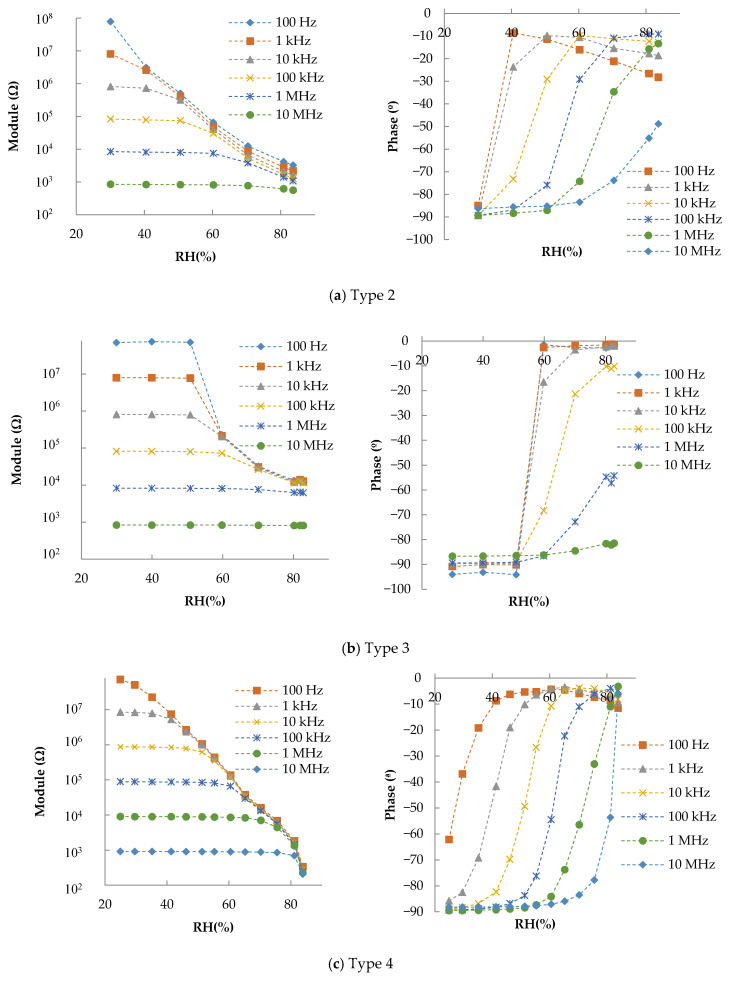

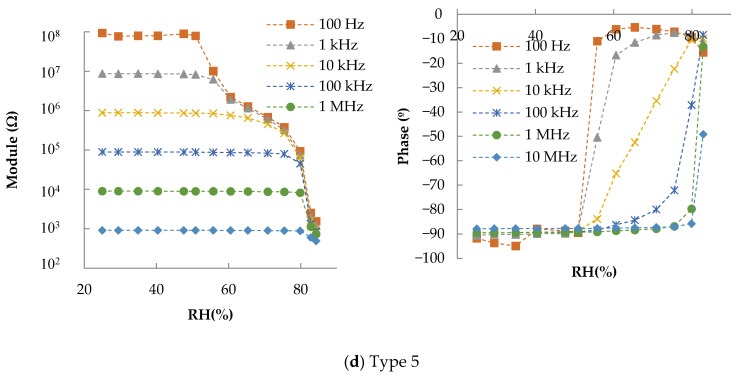
Impedance response towards RH at 40 °C for different operating frequencies for sensors with interspacing of 300 µm: (**a**) type 2; (**b**) type 3; (**c**) type 4; (**d**) type 5. Module is depicted in the left column and phase in the right one.

Figure 3 illustrates the impedance response at 100 Hz for IDEs with an interspacing of 300 µm. The individual frequency response of each device is shown in Figure A1 (see Appendix A). As can be seen, the responses were as expected; the sensor started with a capacitive behavior, and when the RH increased, it changed to a resistive one. In a previous work [20], the device changed its behavior at a RH value of 60%; however, in this work the RH value at which this change occurred varied from 40% to 60% depending on the type of deposition. For deposition types 2 and 4, the trigger value was similar (around 40%), and the same happened with types 3 and 5 which changed around 60%.

As can be appreciated in Figure 2, deposition types 2 and 4 were more uniform than the other two. The drop size was medium, around 50 µm in type 4 and 100 µm in type 2. In the case of type 3 the drops were smaller, around 10 µm and more expatiated. This means that to reach the same conductivity between the IDE fingers, K-Pery needs to be more conductive as there will be less conductive surface area between fingers. In the case of type 5, the drops had a diameter of around 250 µm and big spaces between them. Due to these big spaces, the fingers can contact only one drop or part of them; thus, as occurs with type 3, there is less active surface area between fingers that needs to be compensated for with higher RH values.

Apart from the trigger value, there are other differences in the response of the devices. For example, the type 4 coating response was smoother than others. This behavior can be explained by the different drop density with respect to type 2. The same process seen in type 3 to reach enough conductivity between the IDE fingers happened here; however, as the distribution in type 4 was more uniform and with bigger drops than in type 3, the response was a smoother change instead of a higher trigger value.

**Figure 4:**
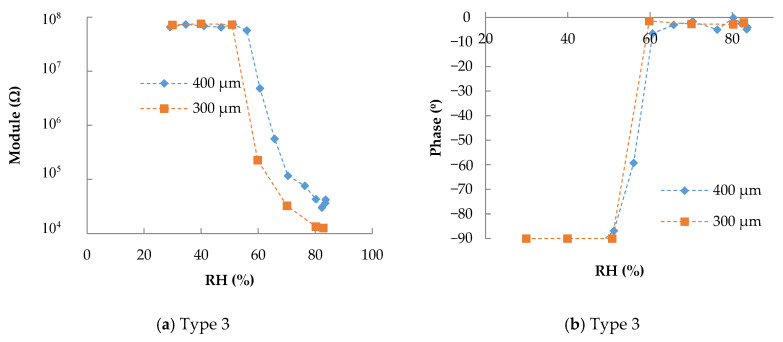

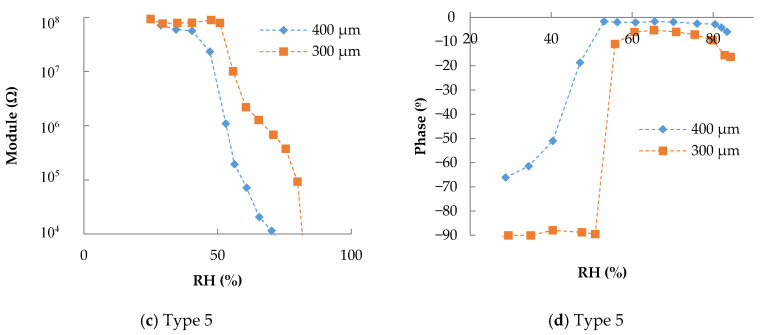
Impedance response towards RH at 100 Hz and 40 °C for different spacing: (**a**) module and (**b**) phase for type 3; (**c**) module and (**d**) phase for type 5.

**Figure A2:**
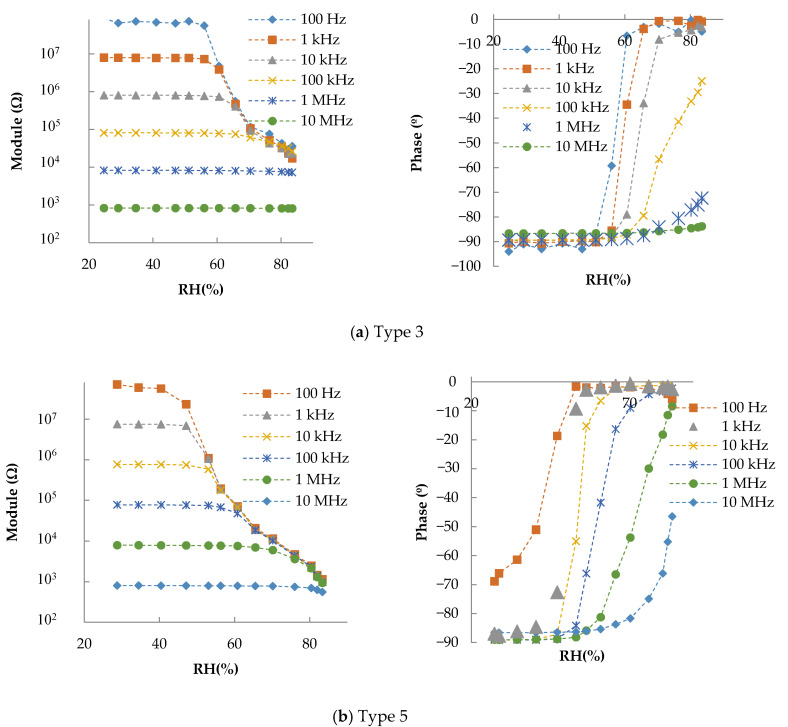
Impedance response towards RH at 40 °C for different operating frequencies for sensors with interspacing of 400 µm: (**a**) type 3 and (**b**) type 5. Module is depicted in the left column and phase in the right one.

The other fabrication parameter that we studied in this work was the interspacing of IDEs. Figure 4 presents the impedance response for type 3 and 5 with interspacing of 300 µm and 400 µm. The finger width was fixed at 200 µm in both cases, and the measuring frequency was 100 Hz. The individual response in frequency is shown in Figure A2 (see Appendix A). In the case of type 3 (Figure 4a,b), the increase in interspacing implies an increase in the trigger value. As happened with higher spaces between drops, with increasing interspacing, the increase in resistance has to be compensated with more RH, so the trigger is at a higher RH value.

In the case of type 5, the interspacing changed the response in the opposite direction, the trigger value was reduced to 50%, and the response was less abrupt than in the other cases.

## 4. Conclusions

Starting from a previous work [20], here two fabrication parameters of K-Pery humidity sensors were analyzed with the objective of being able to control the trigger value of the devices during the fabrication process. The study was centered on the coating parameters and the interspacing of IDEs.

The results show that spray coating allows for the varying of drop sizes and dispersion, which results in different trigger values. These values depend on the amount of material between fingers. As the K-Pery between fingers reduces, bigger RH values are needed to change the state of the device. In this way, we were able to change the trigger value from an RH value of 20% up to a value of 60% depending on the spray parameters used.

The interspacing analysis results showed that the trigger value is also controllable as the increase in the interspace of the IDE reflects an increase in the RH trigger value of the device. By varying this parameter, we were able to change the trigger value; however, the results were not consistent in the different spray parameters tested.

With these results, we demonstrate that K-Pery not only allows for the creation of a humidity actuator, but it is also possible to change the humidity value, which modifies the actuator state, by changing the parameters of the sprayed layer.

## Figures and Tables

**Table 1:** Conditions during fabrication for each of the deposited K-Pery layers.

Name	Fabrication Parameters	
	Volume (mL)	Temperature (°C)
Type 1	2	70
Type 2	2	90
Type 3	2	110
Type 4	4	70
Type 5	4	110

**Figure A3:**
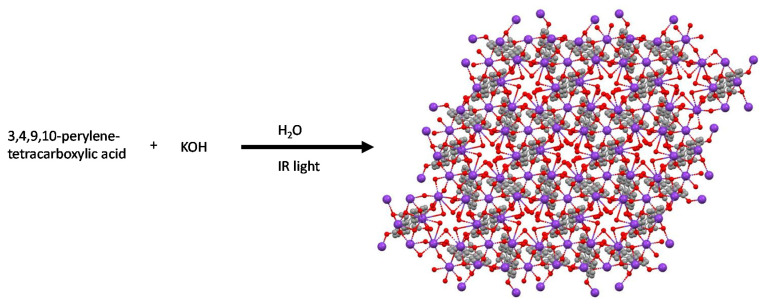
Synthetic scheme with the structure of the MOF.

**Figure A4:**
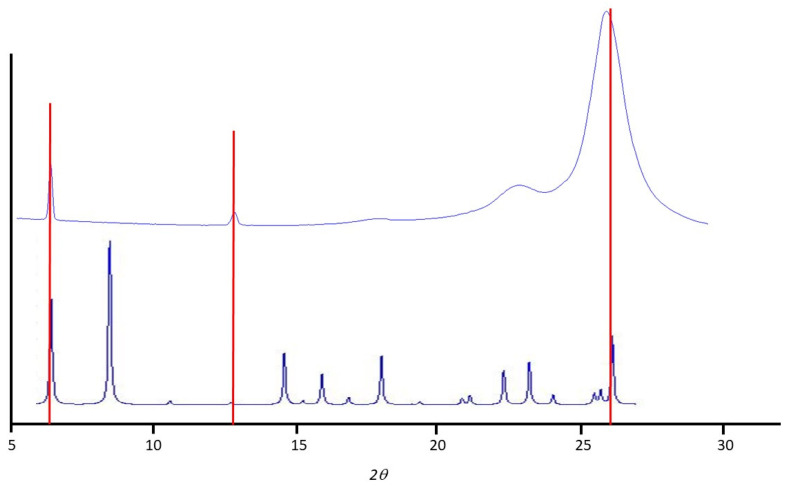
Top: Experimental PXRD of sample after spray coating deposition. Down: Theoretical PXRD obtaining of single crystal data.

## Appendix A

Powder XR Diffraction (PXRD) Analysis.

The powders were gently ground in an agate mortar and then deposited with care in polyethylene terephthalate. Diffraction data (Cu Kα, λ = 1.5418 Å) were collected on a θ:θ Bruker AXS D8 vertical scan diffractometer equipped with primary and secondary Soller slits, a secondary beam curved graphite monochromator, a Na(Tl)I scintillation detector, and pulse height amplifier discrimination. The generator was operated at 40 kV and 40 mA. Optics used were the following: divergence 0.5°, antiscatter 0.5°, receiving 0.2 mm. A long scan was performed with 5 < 2θ < 30° with t = 5 s and ∆2θ = 0.02°.

As can be shown, the main peaks of the experimental measurement coincide with the theoretical peaks of the diffractogram obtained from the crystalline data. Peaks: 6.32 (hkl 001), 12.67 (hkl 002), and 26.09 (hkl 301).
